# Impact of the Aging Lens and Posterior Capsular Opacification on Quantitative Autofluorescence Imaging in Age-Related Macular Degeneration

**DOI:** 10.1167/tvst.11.10.23

**Published:** 2022-10-14

**Authors:** Andreas Berlin, Mark E. Clark, Thomas A. Swain, Nathan A. Fischer, Gerald McGwin, Kenneth R. Sloan, Cynthia Owsley, Christine A. Curcio

**Affiliations:** 1 Department of Ophthalmology and Visual Sciences, School of Medicine, University of Alabama at Birmingham, Birmingham, AL, USA; 2 University Hospital Würzburg, Würzburg, Germany; 3 Department of Epidemiology, School of Public Health, University of Alabama at Birmingham, Birmingham, AL, USA

**Keywords:** age-related macular degeneration (AMD), quantitative fundus autofluorescence (qAF), cataract, pseudophakia, posterior capsule opacification (PCO), retinal pigment epithelium (RPE)

## Abstract

**Purpose:**

The purpose of this study was to investigate quantitative autofluorescence (qAF8) in patients with and without early or intermediate age-related macular degeneration (AMD); to determine the impact of the aged crystalline lens and posterior capsular opacification (PCO).

**Methods:**

In phakic and pseudophakic eyes ≥60 years, AMD status was determined by the Beckman system. PCO presence and severity was extracted from clinical records. qAF8 was calculated using custom FIJI plugins. Differences in qAF8, stratified by lens status, PCO severity, and AMD status, were analyzed using generalized estimating equations.

**Results:**

In 210 eyes of 115 individuals (mean age = 75.7 ± 6.6 years), qAF8 was lower in intermediate AMD compared to early AMD (*P* = 0.05). qAF8 did not differ between phakic and pseudophakic eyes (*P* = 0.8909). In phakic (*n* = 83) and pseudophakic (*n* = 127) eyes considered separately, qAF8 did not differ by AMD status (*P* = 0.0936 and 0.3494, respectively). Qualitative review of qAF images in phakic eyes illustrated high variability. In pseudophakic eyes, qAF8 did not differ with PCO present versus absent (54.5% vs. 45.5%). Review of implanted intraocular lenses (IOLs) revealed that 43.9% were blue-filter IOLs.

**Conclusions:**

qAF8 was not associated with AMD status, up to intermediate AMD, considering only pseudophakic eyes to avoid noisy images in phakic eyes. In pseudophakic eyes, qAF8 was not affected by PCO. Because blue-filter IOLs may reduce levels of exciting light for qAF8, future studies investigating qAF in eyes with different IOL types are needed.

**Translational Relevance:**

To reduce variability in observational studies and clinical trials requiring qAF8, pseudophakic participants without blue-filter IOLs or advanced PCO should be preferentially enrolled.

## Introduction

Age-related macular degeneration (AMD) degrades sight in older adults worldwide,^1^^,^^2^ and involves dysfunction of the retinal pigment epithelium (RPE).^3^ To prevent vision loss, further understanding of RPE health at different AMD stages is sought. A valuable tool for clinically visualizing RPE homeostasis and metabolism is fundus autofluorescence (FAF) imaging, a projection image of all chorioretinal layers.^4^

Quantitative fundus autofluorescence (qAF) uses an internal reference to normalize FAF intensity^5^ and enables comparison of eyes between study groups and over time within individual patients.^6^^–^^8^ Currently used primarily in studies of inherited retinopathies, qAF has revealed disease activity in photoreceptors and RPE associated with specific gene mutations.^9^^–^^13^ Recent qAF studies of early and intermediate AMD eyes have shown a signal similar to or less than that in healthy controls.^14^^,^^15^

The principal subcellular signal source of blue FAF (excitation wavelength, 488 nm) is RPE lipofuscin and melanolipofuscin.^16^^–^^19^ These organelles derived from photoreceptor outer segment tips accumulate in RPE cell bodies starting in childhood,^20^ in a topography precisely linked to the photoreceptors.^18^^,^^21^^–^^23^ Bis-retinoid derivatives of vitamin A are suspected as the fluorophores underlying human FAF.^24^ Increased and decreased FAF signal in AMD are impacted by RPE morphology and organelle content as well as adjacent tissue layers that add to or block the signal.^16^^,^^25^^,^^26^ Expansion of an area of a markedly reduced FAF signal is approved as a clinical trial end point, representing late stage disease.^27^^,^^28^ The idea that an FAF signal might increase in earlier AMD stages was initially supported by model systems and low-spatial resolution human eye pathology (e.g. assays of whole eyecups).^21^ Recent studies showing lipofuscin redistribution and loss as well as stacking and migration of RPE cells in AMD well before atrophy suggests that a decreased or variable signal is likely.^26^

The optical density and autofluorescence of the crystalline lens increase with age and vary from person to person, impacting all fundus imaging and especially blue FAF/qAF imaging.^29^^–^^33^ The aged lens absorbs ultraviolet light and limits its transmission to and from the retina.^5^ How the aged lens influences qAF imaging is currently only partly understood.^34^ To correct the qAF signal for lens opacity, a universal correction factor for age has been established.^5^^,^^6^^,^^29^ However, a single factor may not adequately compensate for wide individual differences of lens opacification.

**Figure 1.**
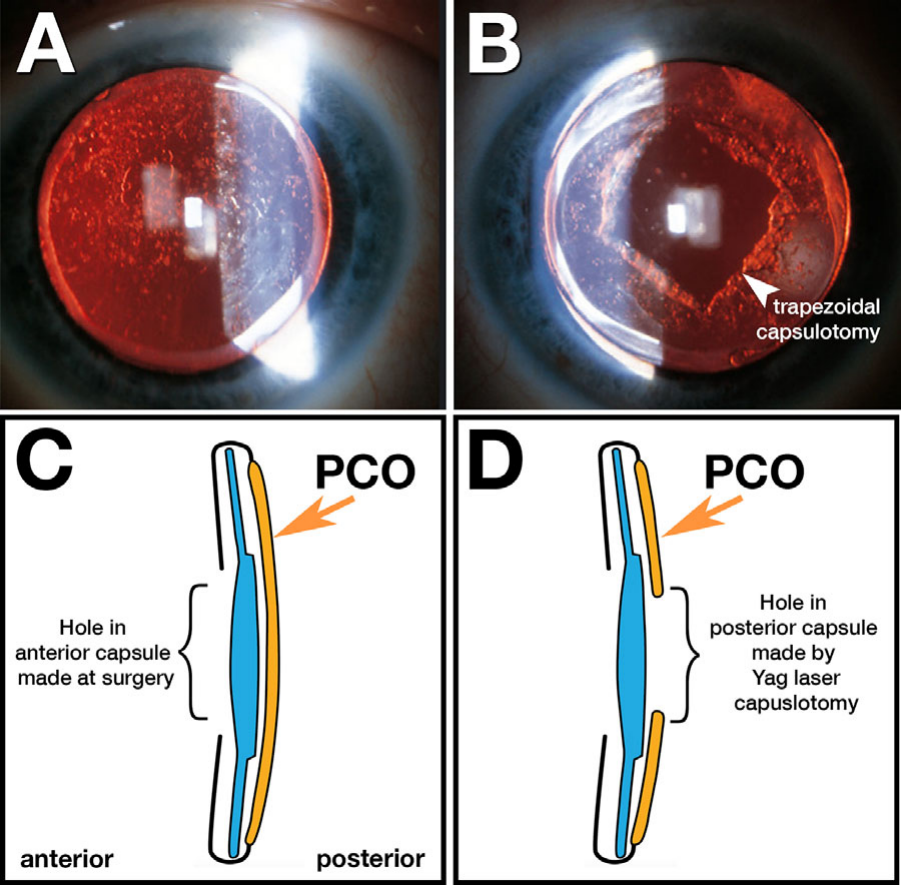
**Posterior capsular opacification following intraocular lens implant.** Posterior capsular opacification (PCO) is corrected with a capsulotomy, performed with a neodymium-doped yttrium aluminum garnet (Nd:YAG) laser. (**A, B**) Reflected light in retro-illumination of the same eye, pre- and post-laser treatment (for illustration purpose only; this eye was not included in the study. This capsulotomy is less than 6 mm diameter. Thereby the remaining opacity might impact the quality of qAF images). (**C, D**) Schematics show a lateral view. **A** and **C** Severe PCO (grade 4) in a pseudophakic 2 years after surgery and before laser capsulotomy. **B** and**D** PCO after laser capsulotomy. **B** The opened PCO is thick at the trapezoidal capsulorhexis margin. **D** The posterior capsule is opened, and some PCO remains. De-identified clinical images courtesy of Arno Sailer, MD, Kolsass, Austria.

In industrialized countries, age-related lens opacity is routinely addressed by cataract extraction and surgical implantation of intraocular lenses (IOLs).^35^^–^^38^ Because half of Americans older than 75 years have IOLs,^39^ it is useful to know if the IOL itself or follow-on procedures affect qAF imaging. IOLs have a range of light transmission properties, and some selectively reduce blue light in addition to ultraviolet light. Further, a complication of cataract surgery, called posterior capsule opacification (PCO), reduces central light transmission and visual acuity in about 28% of eyes after 5 years (Fig. 1).^40^ Fibrotic PCO represents connective tissue transformation of the lens capsule, whereas regenerative PCO represents proliferation of epithelial cells remaining on the capsule surface.^41^ To restore light transmission and acuity, a neodymium: yttrium-aluminum-garnet (Nd:YAG) capsulotomy is currently the gold standard.^41^

Our purpose herein was to investigate perifoveal qAF at 6 degrees to 8 degrees eccentricity (qAF8) in patients with and without early or intermediate AMD. We compared qAF8 in phakic and pseudophakic eyes, and, among pseudophakic eyes, we investigated the impact of PCO.

## Methods

### Compliance

This study was approved by the institutional review board at the University of Alabama at Birmingham (protocol # 170324006). It adhered to the tenets of the Declaration of Helsinki and complied with the Health Insurance Portability and Accountability Act of 1996.

### Study Population

Participants were recruited from comprehensive ophthalmic practices in the Callahan Eye Hospital Clinics during 2017 to 2018, as described.^42^^,^^43^ To be eligible, eyes were required to meet fundus criteria for normal macular health, early AMD, or intermediate AMD. For comparison with existing qAF literature in AMD, eyes were graded using three-field digital stereo color fundus photographs (Carl Zeiss Meditec 450+, Dublin, CA) by an experienced and masked grader (author M.E.C.) using the Beckman classification system.^44^ Previous diagnoses of glaucoma, other retinal conditions, optic nerve conditions, corneal disease, diabetes, Alzheimer's disease, Parkinson's disease, brain injury, and other neurological or psychiatric conditions as revealed by the medical record or by self-report were exclusion criteria.

Demographic characteristics (age, sex, and race/ethnicity) were obtained via participant interview. Lens status was determined by the anterior segment slit lamp photographs (Carl Zeiss Meditec 450+). For IOLs in pseudophakic participants, the manufacturer and model were determined from the medical record. PCO status was determined by slit lamp assessment, as indicated in the clinic electronic health record. PCO status was categorized as “present” and “not present.” “Not present” included either a clear posterior capsule or an open posterior capsule after laser capsulotomy. PCO severity was classified as trace, 1+, 2+, and 3+ in the clinical record, following published grading systems.^45^ Eyes lacking record of PCO status were excluded from the PCO analysis. Ophthalmologic assessments included measurement of corneal curvatures (IOL Master; Carl Zeiss) and best corrected visual acuity using the Electronic Visual Acuity tester (EVA; JAEB Center, Tampa, FL) under photopic conditions (100 cd/m^2^) and expressed as the logarithm of the minimum angle of resolution (logMAR).

### Clinical Image Capture and Analysis

For multimodal imaging, eyes were dilated to a minimum of 6.5 mm pupil diameter using 0.5% tropicamide and 2.5% phenylephrine. Multimodal imaging included qAF, near infrared reflectance (NIR), and spectral-domain optical coherence tomography (OCT; 6-mm horizontal macular scan, 35 frames, 49 B-scans, 20 degrees × 20 degrees field) using a Spectralis device (Heidelberg Engineering, Heidelberg, Germany) modified for qAF as described.^8^ All images were adjusted using participant corneal c-curves for calculation of an individual scaling factor.^6^

Briefly, the Spectralis device contains an internal qAF reference that is excited simultaneously with the fundus (image size = 30 × 30, 768 × 768 pixels, excitation 488 nm and emission = 500–750 nm). In this way, variations in laser power and camera settings between examinations or between subjects can be normalized. To reduce FAF signal attenuation by rod photopigment, photoreceptors were bleached for at least 20 seconds before registration of 12 single FAF gray scale measurement frames.^5^^,^^46^ These frames were immediately checked for homogeneous illumination of the posterior pole and centration of the image on the fovea. Low-quality frames were removed from consideration at this time. The remaining image frames were used to create an average gray scale FAF image using the manufacturer's software. Subjects were excluded from further analysis if fewer than nine frames were useable.

qAF was described by Delori et al.^5^ to analyze gray scale measurements in an averaged AF image. Kleefeldt et al.^8^ extended this approach using custom plugins for FIJI (FIJI Is Just; ImageJ 2.0.0-rc- 69/1.52p; www.fiji.sc; available at: https://sites.imagej.net/CreativeComputation/). qAF in an individual eye represents mean gray value of each pixel relative to that measured through the optical media of an emmetropic eye of a 20-year-old.^5^^,^^6^ The qAF correction for media (cornea, aqueous, lens, and vitreous), in turn, incorporated templates for light absorption based on extensive literature review by van de Kraats and van Norren.^29^

To compare qAF between and within subjects we used qAF8,^5^^,^^6^ defined as mean pixel intensities in a ring of 8 evenly spaced segments, in the perifovea, at 6 degrees to 8 degrees eccentricity. A previous description of 9 degrees to 11 degrees for this location was incorrect.^8^ qAF8 was chosen by its originators to avoid blocking of the signal by macular pigment and to reduce signal noise due to non-autofluorescent vessels at the arcades.^5^^,^^6^ Placement of the qAF8 ring in most prior literature was based on the examiner's visual impression of the position of the fovea and the optic disc. Thus, to standardize anatomic landmarks in a Cartesian coordinate system, we used the “Find Fovea OCT” plugin^8^ on the macular OCT volume and corresponding NIR image, as described.^8^ Within the OCT B-scan, the position of the fovea was selected at the maximal rise of the external limiting membrane (central bouquet)^47^^–^^49^ within the foveal pit. Next, the edge of the optic nerve head closest to the fovea was marked.

We then used the “QAF XML Reader” plugin^8^ to enter the subject's age to compensate for attenuation of qAF signal by age-related media changes.^5^^,^^6^^,^^29^ At this time, a device-specific calibration factor (provided by Heidelberg Engineering) was also entered.^5^^,^^6^ For phakic eyes, the participant's age was entered for a “one-size-fits-all” correction. For pseudophakic eyes, no correction was made, per convention.^5^^,^^6^ qAF images were registered to the NIR image using the “Register OCT” plugin.^8^ qAF was then derived using the “Batch Grids OCT” plugin^8^ and stored in tab-delimited text files for calculation of qAF8 and statistical analysis. Color-coded maps generated from gray scale images were used for qualitative analysis.

### Statistical Analysis

Demographic, best-corrected visual acuity, AMD status and severity, and PCO status were summarized using means and standard deviations or number and percent for continuous and categorical data, respectively. Generalized estimating equations, which account for 2 eyes, were used to compare qAF8 by lens status, AMD status and severity, and PCO status and severity. In addition, each AMD severity category was compared to each other in pairwise comparisons. All models were age adjusted and the level of significance was *P* ≤ 0.05 (2-sided). All analyses were done in SAS version 9.4.

## Results

Of 230 examined eyes, 20 were excluded from analysis due to poor image quality, leaving 210 eyes from 115 individuals (mean age = 75.7 ± 6.6 years, 47 women [40.9%]). Demographic information for participants is summarized in Table 1. Eye-level data are shown in Table 2. These include AMD presence and severity (*n* = 79 [37.6%] normal, 53 [25.2%] early AMD, and 78 [37.1%] intermediate AMD), and lens status (83 phakic and 127 pseudophakic eyes). Phakic and pseudophakic eyes had similar proportions of normal, early AMD, and intermediate AMD eyes (37.4%, 15.7%, and 47.0% vs. 30.7%, 22.8%, and 46.5%, respectively). Among all eyes, the average participant age was 75.3 ± 4.7, 74.0 ± 6.2, and 76.0 ± 7.6 years for those judged with normal, early, and intermediate AMD, respectively. Participants with pseudophakic eyes were older, 77.3 ± 5.9 years, relative to phakic eyes (72.3 ± 6.3). Characteristics of implanted IOLs are displayed in Supplementary Table S1. Information was retrievable for 66 (51%) of 127 pseudophakic eyes. Implanted IOL were mainly monofocal (84.8%) with a few toric (15.2%). Blue light filter IOLs (400-475 nm) were implanted in 29 (43.9%) of 66 eyes.

**Table 1.** Demographic Characteristics of Participants (*N* = 115)

Characteristic	Value
Age, y, mean (SD)	75.7 (6.6)
Age group, *n* (%)	
60–69	19 (16.5)
70–79	69 (60.0)
80–89	23 (20.0)
90–100	4 (3.5)
Gender, *n* (%)	
Male	68 (59.1)
Female	47 (40.9)
Race, *n* (%)	
White	111 (96.5)
African American	3 (2.6)
Asian or Pacific Islander	1 (0.9)

**Table 2.** AMD Status (Beckman Classification System) and Best Corrected Visual Acuity

	All Eyes	Phakic Eyes	Pseudophakic Eyes
	(*N* Eyes = 210)	(*N* Eyes = 83)	(*N* Eyes = 127)
**Characteristic**			
AMD status, *n* (%)			
Normal	79 (37.6)	31 (37.4)	39 (30.7)
Early	53 (25.2)	13 (15.7)	29 (22.8)
Intermediate	78 (37.1)	39 (47.0)	59 (46.5)
**Visual function**			
Best-corrected visual acuity (logMAR), mean (std)	0.09 (0.19)	0.07 (0.13)	0.10 (0.21)

Std, standard deviation.

We first consider the impact of AMD status. Differences in qAF8 among disease severity groups were significant, when the entire sample of eyes was considered (*P* = 0.05; Table 3). Mean qAF8 was higher by 5.2% in early AMD eyes than normal eyes and lower by 18.7% in intermediate AMD eyes than in early AMD eyes. Pairwise comparison of qAF8 by disease status in the entire sample of phakic and pseudophakic eyes showed a significant difference only between early and intermediate AMD (*P* = 0.0152). The pairwise comparisons of normal versus early AMD and normal vs intermediate AMD, in the entire sample of phakic and pseudophakic eyes, were not significant (*P* = 0.2038 and *P* = 0.3678, respectively). Considering either phakic or pseudophakic eyes separately, the qAF8 values did not differ significantly among normal, early, or intermediate AMD in either group (see Table 3).

**Table 3.** Comparison of qAF_8_ Values by AMD Status and Severity Classified Using the Beckman Scales Judged Normal, Early, or Intermediate AMD

Disease status	*n* (%) of Eyes	Mean (std)^c^	*P* Value^b^
**A.** All eyes (*N* persons = 115, *N* eyes = 210)^a^			
			0.0500
Normal	70 (33.3)	228.4 (77.2)	
Early	42 (20.0)	240.1 (71.2)	
Intermediate	98 (46.7)	(71.8)	
**B.** Phakic eyes (*N* persons = 47, *N* eyes = 83)			
			0.0936
Normal	31 (37.4)	241.2 (80.9)	
Early	13 (15.7)	249.3 (49.9)	
Intermediate	39 (47.0)	(81.0)	
**C.** Pseudophakic eyes (*N* persons = 74, *N* eyes = 127)			
			0.3494
Normal	39 (30.7)	218.3 (73.6)	
Early	29 (22.8)	236.0 (79.4)	
Intermediate	59 (46.5)	191.5 (65.4)	

a Pairwise comparison of qAF 8 by disease status in all eyes: normal versus early AMD *P* = 0.2038; normal versus intermediate AMD *P* = 0.3678; early versus intermediate AMD *P* = 0.0152.

b Comparison by AMD status and severity from generalized estimating equations adjusting for age.

c Between phakic and pseudophakic eyes there was no significant difference in qAF8 variability, as assessed by the mean minimum, maximum, and standard deviation of the collected data (*P* values 0.3989, 0.1864, and 0.6385, respectively).

Std, standard deviation.

The impact of natural and implanted IOLs on qAF8 is considered next. Comparing phakic and pseudophakic eyes, no overall difference in qAF8 was detected (mean ± standard deviation: phakic = 223.7 ± 79.1 and pseudophakic = 209.9 ± 73.1, *P* = 0.8909; Table 4). Among pseudophakic eyes, the qAF8 did not differ significantly by PCO status and severity (Table 5), although only 10% of pseudophakic eyes had moderate or advanced PCO (+2 and +3).

**Table 4.** Quantitative Fundus Autofluorescence in all Eyes, Stratified by Lens Status

	All Eyes	Phakic Eyes	Pseudophakic Eyes	
	(*N* Eyes = 210)	(*N* Eyes = 83)	(*N* Eyes = 127)	*P* Value^a^
qAF_8_, mean (std)	215.3 (75.7)	223.7 (79.1)	209.9 (73.1)	0.8909

a Comparison between phakic and pseudophakic eyes from generalized estimating equations adjusting for age.

Std, standard deviation.

**Table 5.** qAF_8_ Stratified by PCO Presence and Severity (*N* Persons = 57, *N* eyes = 99)

PCO Categorization	*n* (%) of Eyes	Mean qAF8 (std)	*P* Value^a^
PCO status and severity			0.3296
Clear	22 (22.2)	217.9 (68.3)	
Trace	8 (8.1)	205.4 (64.6)	
1+	14 (14.1)	202.4 (61.8)	
2+ or 3+	10 (10.1)	196.5 (63.0)	
Open (after laser capsulotomy)	45 (45.5)	214.7 (72.1)	
PCO yes/no, “clear” or “open”			0.1296
Open or clear	67 (67.7)	215.8 (70.4)	
Trace to 3+	32 (32.3)	201.3 (75.2)	

a Comparison by PCO status and severity category from generalized estimating equations.

Std, standard deviation.

**Figure 2.**
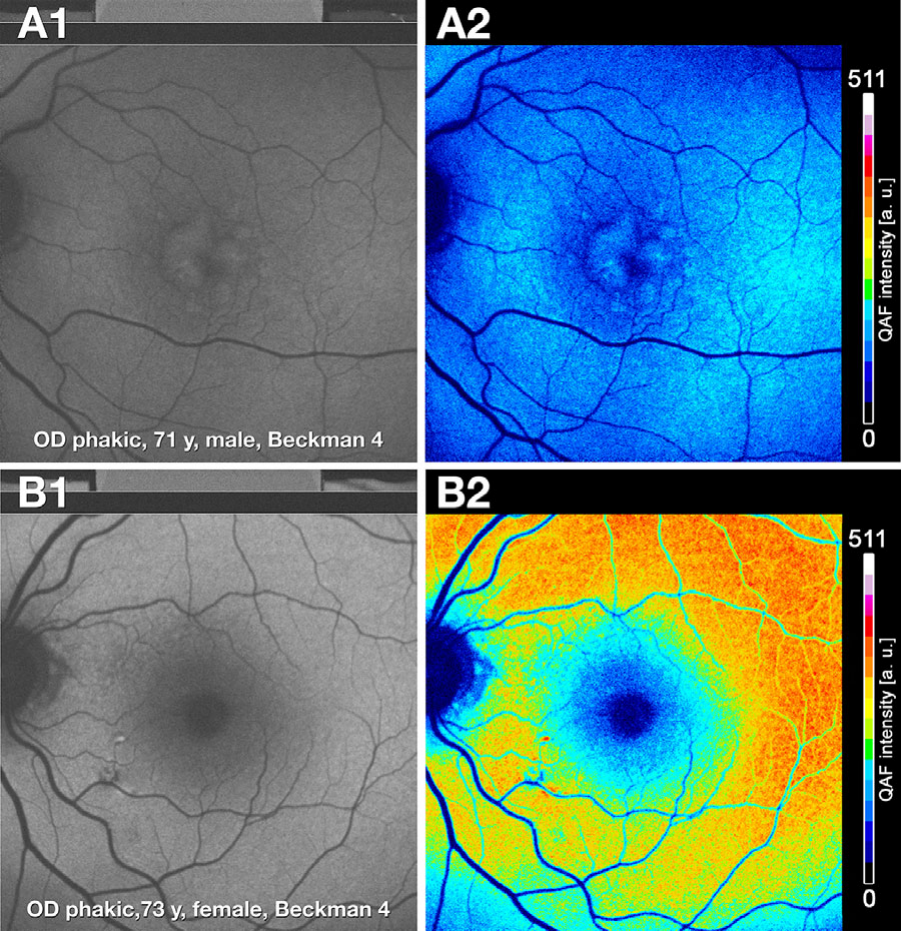
**Impact of crystalline lens on autofluorescence appearance and qAF.** (**A1, A2**) In an aged phakic eye, qAF images are blurred and dim in grayscale and low intensity in color-coded images (**A2**). (**B1, B2**) A comparison eye of similar age and early AMD status shows a less blurred and brighter gray scale qAF image, compared to **A1**. The color-coded qAF image **B2** contains higher and more distinct qAF intensity levels than **A2**. Lens color and opacification grading not available.

**Figure 3.**
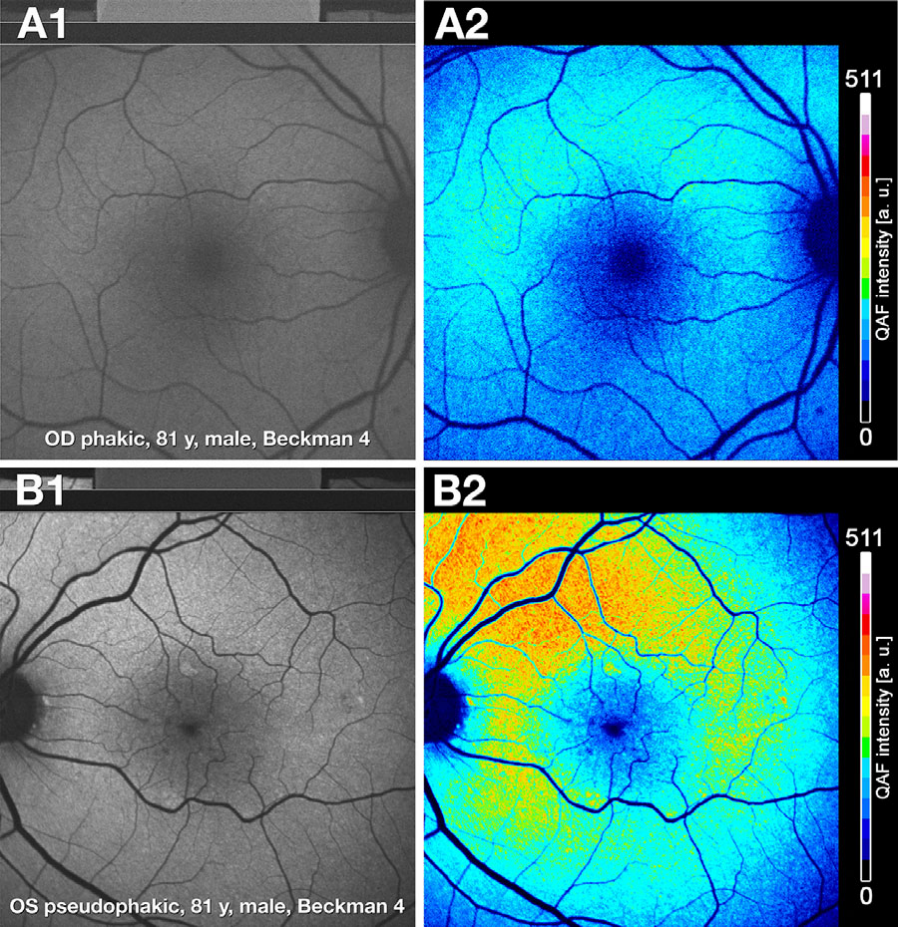
**Quantitative autofluorescence in phakic and pseudophakic fellow eyes.** (**A1, A2**) In an aged phakic eye, qAF grayscale image is blurred and dim, and the color coded qAF image has low intensity **A2**. Lens color and opacification grading not available. (**B1, B2**) In a pseudophakic qAF grayscale image with clear view and high level of detail of the posterior pole in the pseudophakic fellow eye of the same patient as in **A**. The corresponding color coded qAF image **B2** contains higher and more distinct levels of qAF intensity. Both images displayed markedly increased qAF intensity in the superior perifovea.

To elucidate why qAF8 was related to AMD only when phakic and pseudophakic eyes are combined, we next illustrate examples of qAF variability introduced by the aging lens. Figure 2 shows phakic eyes of similar chronologic age. Gray scale images (Figs. 2A1, 2B1) differed in brightness, image focus, and contrast, depending on crystalline lens characteristics. Color-coded qAF values when correctly adjusted for age showed markedly higher qAF intensity in superior and superior-perifoveal regions (Figs. 2A2, 2B2). Figure 3 shows a striking difference between a phakic and pseudophakic eye, at the same AMD severity level, in one participant. In the phakic eye, the qAF gray scale image is blurred and dim (see Fig. 3A1), and the color coded qAF image has low intensity (see Fig. 3A2). In the pseudophakic eye, the qAF grayscale image provides a clear fundus view and displays a high level of detail (see Fig. 3B1). Further, the corresponding color coded qAF image contains higher and more distinct levels of qAF intensity (see Fig. 3B2).

## Discussion

For maximal clinical utility, qAF must address individual variation in the lifespan accumulation of retinal FAF,^6^^,^^8^^,^^50^ lens opacity, and autofluorescence in older persons with crystalline lenses. In pseudophakic eyes, it is possible that the implanted IOL itself, depending on spectral characteristics, or post-surgery PCO will impact light transmission. Our main finding is that qAF8 is lower in intermediate AMD than in early AMD, if phakic and pseudophakic eyes are combined, and not if they are analyzed separately.

Although decreased qAF8 in intermediate AMD comports with our expectations from histology, we currently interpret these results cautiously. First, this decrease was driven by changes in phakic eyes, not pseudophakic eyes. Figures 2 and 3 amply demonstrate the inherent variability introduced by the aging lens. Second, in these phakic eyes, the highest qAF8 occurred in early AMD. In the Beckman grading system, this stage does not include pigmentary changes that might lead to increased signal by rounding or stacking of RPE. Previous qAF studies assessing comparable stages of AMD, also using the Beckman scale^51^^–^^54^ (Table 6), similarly concluded that qAF in early and intermediate AMD does not differ significantly from healthy controls.^14^^–^^15^ Owing to differences in study design, the similar outcomes may be fortuitous. Several reasons may underlie modest or minimal differences between controls and intermediate AMD collectively revealed by qAF8 in the current study and those in Table 6. First, as decreased qAF presages geographic atrophy/complete RPE and outer retinal atrophy (cRORA),^54^^–^^57^ our study eyes may have been positioned too early in AMD progression to register marked qAF8 changes. Second, our model of AMD pathophysiology is deposit-driven end-stages of neovascularization and atrophy, wherein two layers of extracellular deposits (soft drusen and subretinal drusenoid deposit) represent dysregulation of constitutive lipid transfer pathways specialized for cone and rod photoreceptors, respectively.^58^^,^^59^ In this scenario, the location of the qAF8 metric at 6 to 8 degrees eccentricity is not designed to probe the effect of high-risk AMD drusen in the central subfield and inner ring of the ETDRS grid (≤5.2 degrees eccentricity). qAF8 is also not designed to probe areas near the arcades where rod density and AF signal are high and subretinal drusenoid deposits first appear.^21^^,^^22^^,^^60^ Third, qAF may not capture the most relevant predictors of visual decline in AMD. In a recent analysis of spectral domain OCT volumes, retinal locations that were highly predictive of performance on dark adaptation did not involve the RPE cell bodies (containing lipofuscin) but rather, sites on either side of the ellipsoid zone and the RPE-basal lamina-Bruch's membrane band.^61^ The latter may implicate changes in uptake and transfer functions of RPE apical processes and basal infoldings.

**Table 6.** qAF Studies in Early and Intermediate AMD

Author	Demographics (Eyes/Patients)	Lens Status	AMD Groups	Results
Gliem 2016^14^^a^	*n* = 40/40 age = 54.8 ± 5.6 y 108 controls	p	28 SD; 8 CD 4 eAMD; 36 iAMD	no significant qAF8 difference in eAMD-iAMD versus control
Orellana-Rios 2018^53^	*n* = 31/31 age = 83.9 ± 5.39 y 36 controls	pp	17 SD/CD 11 RMD/SDD 8 GA	Mean qAF8 higher in controls than in AMD patients (*P* < 0.001) Significant qAF8 difference SDD versus controls (*P* < 0.05) Lowest mean qAF8 in GA
Reiter 2019^52^^b^	*n* = 88/52 age = 75.6 ± 5.0 y 0 controls	46 (52%) cat^c^ 42 (48%) pp	eAMD, iAMD	No significant association of qAF and drusen volume qAF decreases with age in AMD (*P* = 0.025); drusen volume increases with age
Reiter 2020^51^	*n* = 43/22 age = 73.5 ± 7.9 y 0 controls	24 (56%) cat^c^ 19 (44%) pp	eAMD, iAMD	Excellent repeatability, reliability, follow-up agreement in eAMD and iAMD
Reiter 2021^54^	*n* = 121/71 age = 74.4 ± 5.5 y 0 controls	71 (59%) cat^c^ 50 (41%) pp	121 eyes with iAMD; 21 converted to late AMD	Declining qAF associated with developing atrophic AMD (*P* < 0.001)
Von der Emde 2021^15^	*n* = 85/51 age = 71 ± 7 y 51 controls	68 (80%) p 17 (20%) pp	iAMD	No significant qAF8 difference between AMD eyes with large drusen and healthy eyes (*P* = 0.130) Lower qAF8: pp (*P* = 0.010), male (*P* = 0.008) image quality (*P* = 0.001)
Berlin 2021 (current)	*n* = 210/115 age = 75.7 ± 6.6 y 79 controls	83 (40%) p 127 (60%) pp	53 eAMD, 78 iAMD	No significant difference in qAF8 in p versus pp eyes; PCO presence and severity; normal versus eAMD and iAMD.

cat, cataract; CD, cuticular drusen; eAMD, early AMD; GA, geographic atrophy; iAMD, intermediate AMD; o-c, observational, cross-sectional; o-l, observational longitudinal; p, phakic; PCO, posterior capsular opacification; pp, pseudophakic; SD, soft drusen; RMD, reticular macular disease; SDD, subretinal drusenoid deposits.

All studies used the Beckman Classification System; none of these studies mentioned PCO status

a qAF parameter: horizontal band through the fovea (gray scale histograms along a 3-pixel-wide band).

b qAF parameter: qAFIM (inner and middle ring of Delori grid).

c Cataracts were graded as Lens Opacities Classification System III nuclear ≤3.0 or subcapsular ≤2.0 to be included in the data.

Prior studies (see Table 6) vary as to whether lens opacity and yellowing was assessed.^14^^,^^15^^,^^51^^–^^54^ Age-related lens yellowing from the modification of kynurenine compounds is also responsible for lens autofluorescence.^62^^,^^63^ As the lens opacifies, autofluorescence may be less apparent. Comparison of the same patient pre- and post-cataract surgery has helped define the impact of lens optical properties on imaging.^34^^,^^64^ Reiter et al. demonstrated that reconstituted qAF8 signals after cataract surgery were significantly associated with pre-operative cortical opacity grades.^34^^,^^65^ Although these authors concluded that age-related lens opacities must be incorporated into the interpretation of qAF8, they did not recommend a specific correction.^34^

The literature reveals different ways that investigators mitigated the effect of lens aging in FAF-based retinal imaging. First, restricting study participant age to less than 60 years substantially avoids age-related lens opacification.^5^ A second approach is to objectively grade the extent of age-related changes and set a threshold for data exclusion.^15^^,^^56^^,^^65^ Third, it is possible to use excitation wavelengths longer than 488 nm to bypass short wavelength absorbers in the lens, an approach requiring a separate instrument in most cases.^66^ Longitudinal data on individual eyes can potentially obviate some of the lens effects, because each patient serves as his or her own control over the observation period. For example, Von der Emde et al. reported a lack of significant differences in qAF8 between eyes with and without AMD and declining qAF over time only in the eyes with AMD.^15^ Reiter et al. found declining qAF over time, especially in eyes that converted to atrophic AMD.^54^ We should recall that both lens and RPE may be changing at different rates over the same period. Clearly, a reliable means to correct for lens opacity and autofluorescence on an individual basis would be an important step forward for retinal FAF imaging.

We were surprised to learn that 43% of IOLs in pseudophakic patients in our study were of the type that selectively reduce transmission of blue light. The number of eyes for which lens type could be determined was small, and some surgeries were done years prior to qAF imaging. Prior qAF imaging studies (see Table 6) did not mention blue-filter IOLs among pseudophakic eyes in their samples. Eyes with blue-blocking IOLs may impact qAF imaging by reducing a small proportion of light at the excitation wavelength of 488 nm.^67^ Determining how much reduction is of importance, but it was beyond the scope of this study, which was retrospective with regard to the IOL inquiry. Prior studies (see Table 6) also did not consider the effect of PCO in pseudophakic eyes on qAF imaging.^14^^,^^15^^,^^51^^–^^54^ Our data thus add novelty to existing literature by suggesting that mild or moderate PCO status does not affect qAF values.

Strengths of our study are qAF8 values from the largest number of normal aged eyes (*n* = 70) and pseudophakic eyes (*n* = 127) to date, novel findings on PCO, and perspective from recent clinical and laboratory findings in human eyes. Limitations include a relatively small sample, and a very small number of eyes with advanced PCO. Further, PCO status was obtained retrospectively via electronic health record review, and no information about the capsulotomy technique and size was available. Age changes of the crystalline lens were not objectively graded. IOL characteristics were available for only half of the pseudophakic eyes, and these were heterogeneous with respect to optic type and transmission spectrum.

Despite these limitations, our analysis suggests that qAF8 is minimally affected up to intermediate AMD, changes in phakic eyes are due to lenticular effects, and qAF8 in pseudophakic eyes is not affected by the presence of moderate PCO. To maximize the clinical utility of qAF, future studies investigating qAF in eyes with different IOL types are needed. In the meantime, we recommend that studies assessing qAF8 in aged patients enroll only pseudophakic eyes without blue-filter IOLs and advanced PCO, to reduce variability. The lack of significant qAF8 differences along the progression from normal to early and intermediate AMD should be confirmed in a larger sample, such as our ongoing prospective observational study.

We draw five broader conclusions from these data. First, fundus AF remains an excellent imaging technology for assessing outer retinal integrity and health in early stages of AMD. Second, qAF offers advantages for comparative studies that can be improved by correction for lenses on an individual basis. Third, qAF8 is a less-than-ideal metric for AMD, because it does not assess sites of key pathology. Fourth, qAF measured at selected locations, at a spatial scale comparable to histology,^8^^,^^17^^,^^68^^,^^69^ and in reference to a normative database,^8^ has potential that should be explored further. Finally, as reviewed,^70^ the value of blue-blocking IOLs for preventing AMD is questionable, and they permanently reduce useful spectrum for rod-mediated vision.^71^^,^^72^ Whether they also impact the utility of FAF imaging for early stages of AMD needs further research.

## Supplementary Material

Supplement 1
