# Supplementary material for: Impact of the Aging Lens and Posterior Capsular Opacification on Quantitative Autofluorescence Imaging in Age-Related Macular Degeneration
Source: Transl Vis Sci Technol. 2022 Oct 14;11(10):23. doi: 10.1167/tvst.11.10.23 (PMC9586138; doi:10.1167/tvst.11.10.23)
Supplement: Supplement 1 [file tvst-11-10-23_s001.pdf]

**Supplementary table 1**

Intraocular lens (IOL) characteristics

| Characteristic | pseudophakic eyes with available information (n = 66 of 127) |
|---|---|
| **Optic type, n (% of 66)** | |
| monofocal | 56 (84.8) |
| toric | 10 (15.2) |
| | |
| **Blue light filter,** n (% of 66)** | |
| all IOL with filter | 29 (43.9) |
| monofocal | 20 (30.3) |
| toric | 9 (13.6) |
| all IOL without filter | 37 (56.1) |
| monofocal | 36 (54.5) |
| toric | 1 (1.5) |

*All IOL were acrylic lenses

** wavelength spectrum of 400-475 nm
